# Conformational exchange of aromatic side chains characterized by L-optimized TROSY-selected ^13^C CPMG relaxation dispersion

**DOI:** 10.1007/s10858-012-9656-z

**Published:** 2012-07-26

**Authors:** Ulrich Weininger, Michal Respondek, Mikael Akke

**Affiliations:** Department of Biophysical Chemistry, Center for Molecular Protein Science, Lund University, P.O. Box 124, 22100 Lund, Sweden

**Keywords:** Relaxation dispersion, Conformational exchange, Protein folding, Aromatic side chain, TROSY

## Abstract

Protein dynamics on the millisecond time scale commonly reflect conformational transitions between distinct functional states. NMR relaxation dispersion experiments have provided important insights into biologically relevant dynamics with site-specific resolution, primarily targeting the protein backbone and methyl-bearing side chains. Aromatic side chains represent attractive probes of protein dynamics because they are over-represented in protein binding interfaces, play critical roles in enzyme catalysis, and form an important part of the core. Here we introduce a method to characterize millisecond conformational exchange of aromatic side chains in selectively ^13^C labeled proteins by means of longitudinal- and transverse-relaxation optimized CPMG relaxation dispersion. By monitoring ^13^C relaxation in a spin-state selective manner, significant sensitivity enhancement can be achieved in terms of both signal intensity and the relative exchange contribution to transverse relaxation. Further signal enhancement results from optimizing the longitudinal relaxation recovery of the covalently attached ^1^H spins. We validated the L-TROSY-CPMG experiment by measuring fast folding–unfolding kinetics of the small protein CspB under native conditions. The determined unfolding rate matches perfectly with previous results from stopped-flow kinetics. The CPMG-derived chemical shift differences between the folded and unfolded states are in excellent agreement with those obtained by urea-dependent chemical shift analysis. The present method enables characterization of conformational exchange involving aromatic side chains and should serve as a valuable complement to methods developed for other types of protein side chains.

**Electronic supplementary material:**

The online version of this article (doi:10.1007/s10858-012-9656-z) contains supplementary material, which is available to authorized users.

Conformational transitions are intimately tied to protein function. Numerous reports have shown that transiently populated high-energy states play important roles in enzyme catalysis (Cole and Loria 2002; Eisenmesser et al. 2002; Sprangers et al. 2005; Boehr et al. 2006) or ligand binding by conformational selection (Malmendal et al. 1999; Brüschweiler et al. 2009). Intermittent transitions between different conformations generally lead to modulation of NMR parameters, such as the chemical shift (Gutowsky and Saika 1953) or residual dipolar couplings (Igumenova et al. 2007; Vallurupalli et al. 2007), resulting in exchange contributions to transverse relaxation rates. Biologically relevant exchange correlation times often fall in the range of milliseconds, which can be probed by NMR relaxation dispersion methods, such as the *R*_1ρ_ (Akke and Palmer 1996) or Carr-Purcell-Meiboom-Gill (CPMG) experiment (Carr and Purcell 1954; Meiboom and Gill 1958) and variants thereof (Loria et al. 1999a, b; Igumenova and Palmer 2006). These experiments provide a powerful means of characterizing exchange processes in terms of the exchange rate, *k*_ex_, the relative populations of the exchanging states, *p*_i_, and the difference in chemical shift, Δω, or residual dipolar coupling between them. In addition to the applications mentioned above, relaxation dispersion methods have proven very successful in studying protein folding, including characterization of transition states as well as intermediate states (Grey et al. 2006; Neudecker et al. 2006; Teilum et al. 2006b; O’Connell et al. 2009; Korzhnev et al. 2010). To date, experiments have been designed to probe conformational exchange at specific sites in proteins, including the backbone (Akke and Palmer 1996; Loria et al. 1999a, b; Hill et al. 2000; Mulder and Akke 2003; Lundström and Akke 2005a, b; Igumenova and Palmer 2006; Lundström et al. 2008, 2009a) and side-chain aliphatic (Lundström et al. 2009b; Hansen et al. 2012), carbonyl/carboxyl (Paquin et al. 2008; Hansen and Kay 2011), and methyl groups (Mulder et al. 2002; Brath et al. 2006; Baldwin et al. 2010; Otten et al. 2010).

Aromatic residues are prevalent in protein binding interfaces, where they contribute significantly to the binding free energy (Bogan and Thorn 1998; Lo Conte et al. 1999; Birtalan et al. 2010). His and Tyr also play prominent roles in enzyme catalysis (Bartlett et al. 2002). Furthermore, aromatic side chains make up a significant proportion of the protein interior, and therefore provide an attractive means of probing the dynamics of the hydrophobic core in the native state (Wüthrich and Wagner 1975), as well as the formation of the core in protein folding reactions. Thus, there is great incentive to extend the existing repertoire of CPMG-type experiments to probe also aromatic side chains in proteins.

We have previously introduced a method for fractional (50 %), site-specific ^13^C labeling of proteins using 1-^13^C_1_-glucose or 2-^13^C_1_-glucose, which produces samples with isolated ^13^C spins thereby eliminating unwanted relaxation pathways and coherent magnetization transfer via one-bond couplings (Teilum et al. 2006a; Lundström et al. 2007). Here, we present a sensitivity-enhanced CPMG relaxation experiment for measuring millisecond conformational exchange of aromatic side chains in selectively ^13^C labeled proteins, based on longitudinal- and transverse-relaxation optimized spectroscopy, denoted L-TROSY (Pervushin et al. 1998; Loria et al. 1999b; Pervushin et al. 2002; Eletsky et al. 2005; Weininger et al. 2012). Figure 1 outlines the pulse sequence for the L-TROSY-CMPG relaxation dispersion experiment for aromatic ^13^C spins.

**Fig. 1:**
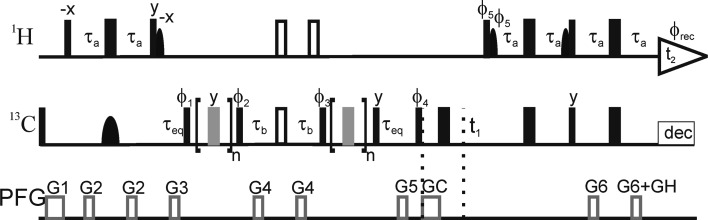
Pulse sequence of the L-TROSY-CPMG relaxation dispersion experiment for measuring conformational exchange of aromatic side chains in specifically ^13^C labeled proteins. All pulses are applied along the x-axis unless otherwise indicated.* Narrow (wide) solid bars* indicate rectangular high-power 90° (180°) pulses.* Wide open bars* indicate composite 180° pulses. *Wide grey bars* indicate 180° pulses in the CPMG elements, which have attenuated power (by 6 dB). *Solid semi-ellipses* represent shaped pulses. *Narrow semi-ellipses* on ^1^H are 90° EBURP2 shapes (Geen and Freeman 1991) centered at 1.9 ppm with a bandwidth of 6.6 ppm. The wide semi-ellipse on ^13^C represents a 180° REBURP pulse (Geen and Freeman 1991) with a bandwidth of 40 ppm. ^13^C is decoupled during acquisition using GARP (Shaka et al. 1985). The delays τ_a_, τ_b_ and τ_eq_ are set to 1.5, 1.4 and 5 ms, respectively. The pulses flanking the CPMG blocks purge non-refocused magnetization remaining as a consequence of the variation among aromatic sites in the ^1^J_HC_ coupling constant (Vallurupalli et al. 2007). The magnetizations from water and aliphatic ^1^H spins are aligned along the +z axis whenever possible, including the CPMG blocks. The phase cycle is: ϕ_1_ = 4(x), 4(−x), ϕ_2_ = (y, −y), ϕ_3_ = (x, −x), ϕ_4_ = (y, x, −y, −x), ϕ_5_ = (−y), ϕ_rec_ = (x, −y, −x, y, −x, y, x, −y). Pulsed field gradients G1–6 are employed to suppress unwanted coherences and artifacts, while GC and GH are encoding and decoding gradients, respectively, for echo/anti-echo coherence selection, obtained by inverting the signs of ϕ_5_, GC and the even-numbered phases of the receiver (Palmer et al. 1991; Kay et al. 1992). Gradient durations (in ms) and power levels (G/cm) are set to (duration, power level): G1 = (1.0, 10); G2 = (0.5, 8); G3 = (0.5, 14); G4 = (0.5, 16); G5 = (0.5, –24); G6 = (0.5, 18); GC = (1.0, 54); GH (0.5, 27.018). For every second t_1_ increment, ϕ_4_ and the receiver were incremented

We applied the L-TROSY-CPMG experiment to characterize fast folding–unfolding of cold-shock protein B (CspB) from *Bacillus subtilis* (Schindler et al. 1995) under native conditions. CspB was expressed in *Escherichia coli* cultured on medium containing 1-^13^C_1_-glucose as the sole carbon source, resulting in enrichment of ^13^C into the δ positions of Phe, δ1 and ε3 of Trp, and δ2 and ε1 of His. CspB contains 7 Phe, 1 Trp, 1 His, but no Tyr.

Figure 2 shows the relaxation dispersion curves acquired using the L-TROSY-CPMG experiment for ^13^C-labeled aromatic side chains. As observed from the comparison with the corresponding data obtained using a regular (i.e. non-L-TROSY) relaxation-compensated (rc) CPMG experiment (Loria et al. 1999a), TROSY-selection achieves significant reduction of the natural linewidth, resulting in increased precision of the measured transverse relaxation rates and vastly improved dynamic range of the relaxation dispersion; see inset of Fig. 2a and Fig. S1. The TROSY effect is expected to be optimal at static magnetic field strengths of approximately 14–15 T, as calculated based on the chemical shielding anisotropy of benzene (Veeman 1984). At 11.7 T, the transverse relaxation rate of the TROSY line is approximately 30 % of the transverse ^13^C autorelaxation rate. TROSY is expected to provide sensitivity enhancement for larger proteins with a rotational correlation time of *τ*_c_ ≥ 13 ns (Weininger et al. 2012). CspB has *τ*_c_ = 4.4 ns at 25 °C, implying that in this case TROSY does not provide any sensitivity enhancement in and of itself. However, longitudinal relaxation optimization (Pervushin et al. 2002; Eletsky et al. 2005; Weininger et al. 2012) offers significant sensitivity enhancement per unit time, already for a small protein like CspB. In particular, ^13^C sites located nearby ^1^H spins that exchange with solvent, e.g. δ1 of Trp or δ2 and ε1 of His, reach gains in signal-to-noise of up to 50 % in the case of CspB. This enhancement is greater than that (10–35 %) documented previously for the larger protein Gal3C (Diehl et al. 2010; Weininger et al. 2012), presumably due to the greater extent of solvent exposure of the aromatic residues in CspB compared to Gal3C. Sensitivity enhancement by L-optimization is expected to increase progressively with molecular weight and static magnetic field strength. As previously described in detail, careful control of the water and aliphatic magnetizations is required in order to obtain accurate relaxation data and optimal sensitivity enhancement using L-optimized experiments (Weininger et al. 2012). In the pulse sequence presented here (Fig. 1), water and aliphatic ^1^H magnetizations are aligned along +*z* during the CPMG, *t*_1_ evolution, and acquisition periods.

**Fig. 2:**
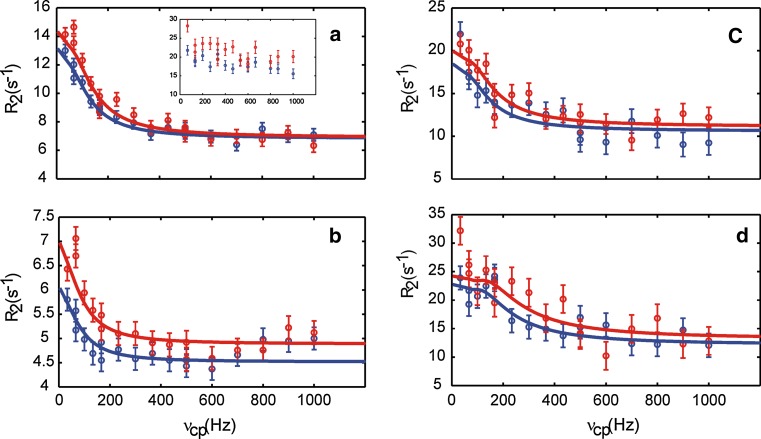
Representative ^13^C aromatic L-TROSY-CPMG relaxation dispersion profiles acquired on a 0.4 mM sample of CspB in 10 mM HEPES pH 7.0 at 25 °C and static magnetic field strengths of 11.7 T (*blue*) and 14.1 T (*red*). Data are shown for residues F15δ* (a), F38δ* (b), W8δ1 (c) and H29ε1 (d). The inset in* panel a* shows the corresponding data acquired with the rc-CPMG sequence. The solid lines in (a–d) represent global fits of the folding–unfolding model to the experimental data. Relaxation delays where chosen so as to get the same signal decay (about 50 %) in all experiments, resulting in delays of 60 ms (L-TROSY-CPMG) or 30 ms (rc-CPMG). Supplementary Information Figure S1 shows results for all residues, including rc-CPMG data and L-anti-TROSY-CPMG data

Significant dispersion profiles were obtained for 9 out of 11 aromatic residues in CspB. The data are adequately represented by a global two-state (folded/unfolded) model using the Carver-Richards equation (Carver and Richards 1972; Palmer et al. 2001), with *k*_ex_ = 528 ± 52 s^−1^ and a population of the unfolded state of *p*_U_ = 2.3 ± 0.2 % (Fig. 2a–d). By contrast, the relaxation data obtained using the rc-CPMG sequence (Fig. 2a, inset; Fig. S1) suffer severely from the fast transverse relaxation of the averaged ^13^C doublet (roughly a factor of two greater than that of the spin-state selective TROSY line), and are clearly not of sufficient quality to warrant further interpretation.

In the context of the L-TROSY-CPMG experiment, it is critical to recognize that the decay of antiphase coherences (e.g. 2CyHz) can be affected by strong coupling between the ^1^H spin covalently attached to the monitored ^13^C spin and neighboring ^1^H spins, which makes the effective *R*_2_ rates depend on the refocusing frequency *ν*_cp_ also in the absence of conformational exchange (van Ingen et al. 2009). In the case of aromatic spin systems, such aberrant dispersions can become significant if strong scalar coupling occurs between vicinal ^1^H spins (e.g. ^1^Hδ1 and ^1^Hε1 in Phe) for which ^3^ *J*_HH_ ≈ 8 Hz, whereas weaker coupling constants (e.g. ^4^ *J*_HH_ ≈ 2 Hz, across the ring) do not cause any significant problems irrespective of the frequency difference between the coupled ^1^H spins, in agreement with previous results (van Ingen et al. 2009). His δ2 and ε1 and Trp δ1 ^1^H spins are always weakly coupled to their vicinal ^1^H neighbors, which are covalently attached to nitrogen and therefore resonate far downfield. In the case of Phe and Tyr residues, rapid ring flipping makes it impossible to determine a priori whether the weak coupling limit applies. However, numerical simulations using QSim (Helgstrand and Allard 2004) demonstrate that artifactual dispersion decays caused by strong scalar coupling level out completely for *ν*_cp_ ≥ 100 Hz, where the ^13^C–^1^H scalar interaction is effectively decoupled. Furthermore, the artifactual dispersion magnitudes are generally limited to *R*_2_(*ν*_cp_ = 0)−*R*_2_(*ν*_cp_ → ∞) ≤3 s^−1^. These two criteria serve as useful guidelines for establishing the accuracy of relaxation dispersions measured for Phe and Tyr.

In CspB, H29ε1 and W8δ1 both show dispersion steps of approximately 10 s^−1^ (Fig. 2c–d). Two out of seven Phe exhibit dispersions of 4–6 s^−1^ (as exemplified by F15δ, Fig. 2a), which also can be safely attributed to conformational exchange. Three remaining Phe rings show smaller dispersion steps of 2 s^−1^ (as exemplified by F38δ, Fig. 2b), and 2 rings do not show any appreciable relaxation dispersion. All aromatic ^13^C dispersion curves of CspB reach a plateau at *ν*_cp_ > 200 Hz, clearly indicating that the dispersions arise from conformational exchange, rather than from effects of strong coupling. Thus, we conclude that the present data are unhampered by any strong coupling between protons and accurately reflect exchange between folded and unfolded states.

The unfolding rate, *k*_U_ = 12 ± 3 s^−1^ (derived from the fitted parameters: *k*_U_ = *k*_ex_·*p*_U_) matches perfectly with that (12 ± 7 s^−1^) determined previously from stopped-flow data (Schindler et al. 1995), whereas the folding rates differ by a factor of two. Notably, it has been observed for CspB that *k*_U_ is almost independent of urea concentration, whereas the folding rate (*k*_F_) exhibits a strong urea dependence (Schindler et al. 1995), indicating that the former is considerably more robust with respect to variations in solvent conditions, such as the difference in buffer composition between the present (10 mM HEPES) and previous (20 or 100 mM sodium cacodylate; Schindler et al. 1995; Zeeb and Balbach 2005) experiments. At the level of individual ^13^C sites, no deviations from two-state folding behavior were observed, in agreement with previous results (Schindler et al. 1995; Zeeb and Balbach 2005). In the present case, ring flips are expected to be significantly faster than the folding–unfolding kinetics. Consequently, the two processes are time-scale separated, such that CPMG dispersion measurements report only on the folding–unfolding process, while exchange due to ring flips in CspB is too fast to be probed by current refocusing rates.

We assessed the accuracy of the chemical shift differences between the folded and unfolded states determined from the CPMG dispersions by comparing with shift differences measured from ^1^H–^13^C HSQC spectra of the folded and unfolded states. ^13^C chemical shifts of the unfolded state under native conditions were obtained by monitoring the urea-dependence of the ^1^H–^13^C HSQC spectrum from 0.5 to 2.5 M urea and extrapolating linearly to 0 M (Fig. S2). Residue-specific assignments of Phe ^13^Cδ resonances in the unfolded state were not necessary, because they all merged to a single overlapped signal.

Figure 3 shows the excellent agreement of the chemical shift differences extracted from the global fit to the CPMG dispersion data, compared to the shift differences between the folded and unfolded states derived from ^1^H–^13^C HSQC spectra. These results demonstrate that aromatic ^13^C chemical shift differences between ground (e.g. folded) and high-energy (e.g. unfolded) states can be determined robustly from CPMG dispersion experiments, similar to what has been reported previously for other types of nuclei (Teilum et al. 2006b; Hansen et al. 2008).

**Fig. 3:**
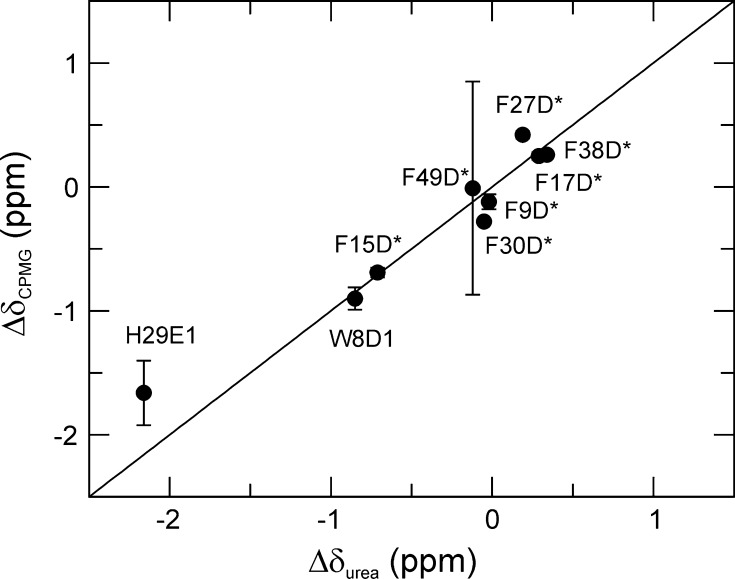
Correlation of ^13^C chemical shift differences, Δδ = Δω/(2πγB_0_), between the folded and unfolded states of CspB derived from L-TROSY-CPMG relaxation dispersions under native conditions or measured directly from ^1^H–^13^C HSQC spectra of the native and progressively urea-denatured states. The signs of the shift differences measured from spectra were also used for the CPMG-derived shift difference. Standard errors of the fitted parameters (vertical axis) were determined from the covariance matrix, whereas the uncertainties of the HSQC-derived shift differences are negligibly small. Sizeable uncertainties are observed only for F49δ*, which has a chemical shift difference close to zero, and H29ε1, which has low signal intensity (Fig. S1); for other residues the error bars are smaller than, or similar to, the size of the symbols

In conclusion, the L-TROSY-CPMG relaxation dispersion experiment for aromatic ^13^C spins provides accurate information on conformational exchange, including the aromatic chemical shifts of the transiently populated high-energy state, and should serve as a valuable complement to experiments developed for other types of side chains.

## Electronic supplementary material

Below is the link to the electronic supplementary material.

SUPPLEMENTARY MATERIAL: One figure showing relaxation dispersion curves for all data acquired on CspB using L-TROSY-CPMG and rc-CPMG experiments. One figure showing the urea dependence of the ^13^C chemical shifts of the unfolded state of CspB at 25°C. (PDF 1591 kb)
