# Supplementary material for: Conformational exchange of aromatic side chains characterized by L-optimized TROSY-selected 13C CPMG relaxation dispersion
Source: J Biomol NMR. 2012 Jul 26;54(1):9–14. doi: 10.1007/s10858-012-9656-z (PMC3427480; doi:10.1007/s10858-012-9656-z)
Supplement: Supplementary file 1 — SUPPLEMENTARY MATERIAL: One figure showing relaxation dispersion curves for all data acquired on CspB using L-TROSY-CPMG and rc-CPMG experiments. One figure showing the urea dependence of the 13C chemical shifts of the unfolded state of CspB at 25°C. (PDF 1591 kb) [file 10858_2012_9656_MOESM1_ESM.pdf]

Supporting Information

Conformational Exchange of Aromatic Side Chains Characterized by L-optimized TROSY-Selected ^{13}C CPMG Relaxation Dispersion

Ulrich Weininger, Michal Respondek, and Mikael Akke*

Department of Biophysical Chemistry, Center for Molecular Protein Science, Lund University, P.O. Box 124, SE-22100 Lund, Sweden

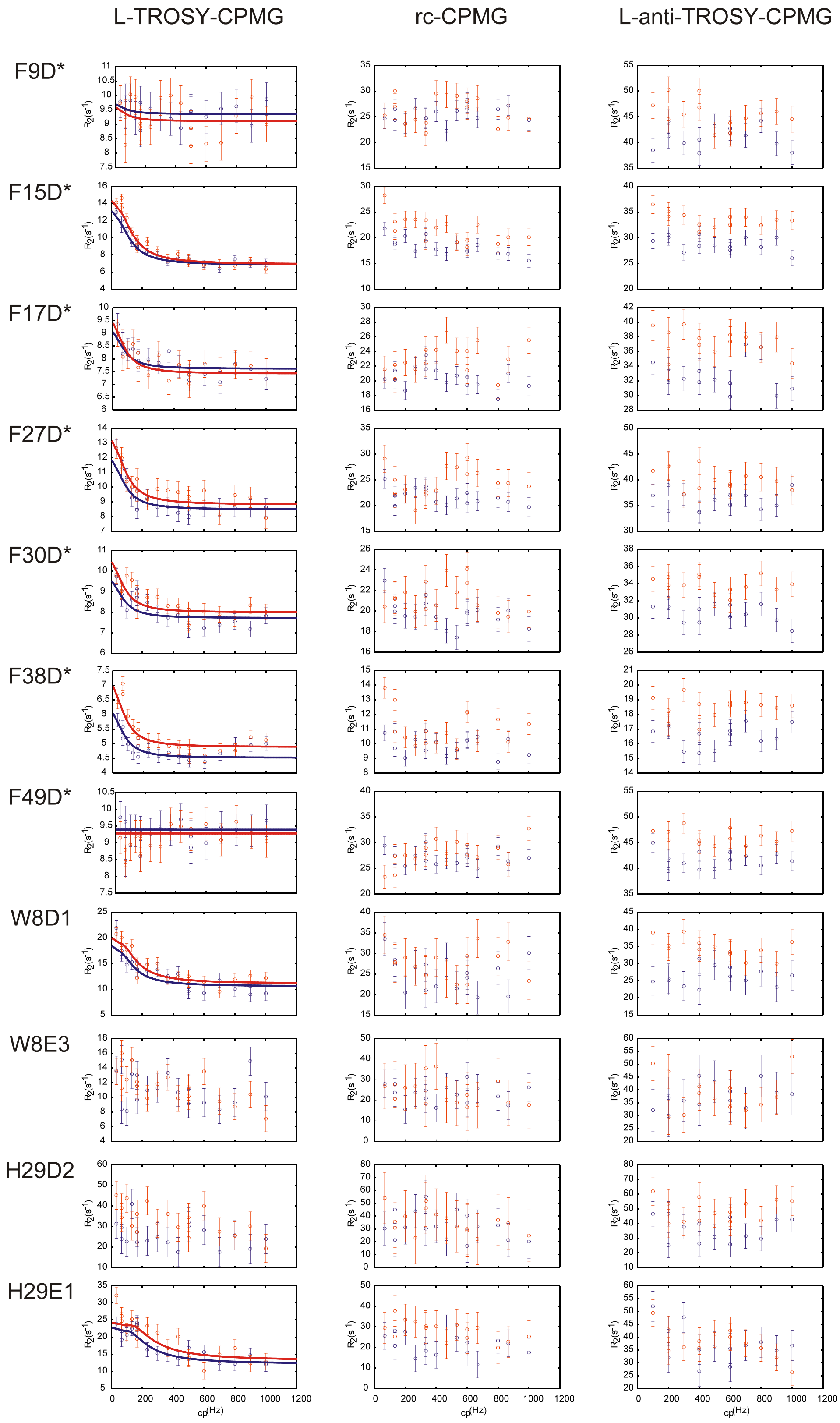

SI Figure 1. All obtained ^{13}C aromatic CPMG relaxation dispersion profiles in three different versions acquired on a 0.4 mM sample of CspB in 10 mM HEPES pH 7.0 at 25 °C and static magnetic field strengths of 11.7 T (blue) and 14.1 T (red). The solid lines in the L-TROSY variant represent global fits of the folding–unfolding model to the experimental data.

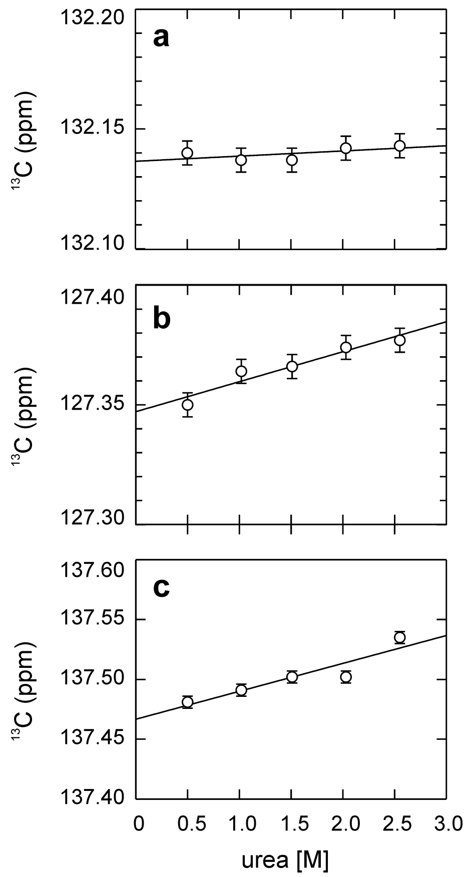

SI Figure 2. Urea dependence of the ^{13}C chemical shifts of the unfolded state of CspB at 25°C monitored by ^{1}H-^{13}C HSQC. (a) Phe D*, (b) Trp D1, (c) His E1.
